# Supplementary material for: The Construction and Exploration of a Comprehensive MicroRNA Centered Regulatory Network in Foxtail Millet (Setaria italica L.)
Source: Front Plant Sci. 2022 May 6;13:848474. doi: 10.3389/fpls.2022.848474 (PMC9121102; doi:10.3389/fpls.2022.848474)
Supplement: Supplementary file 7 [file Data_Sheet_7.zip › Sit-miRN3027_Seita.5G043600.1_1459_TPlot.pdf]

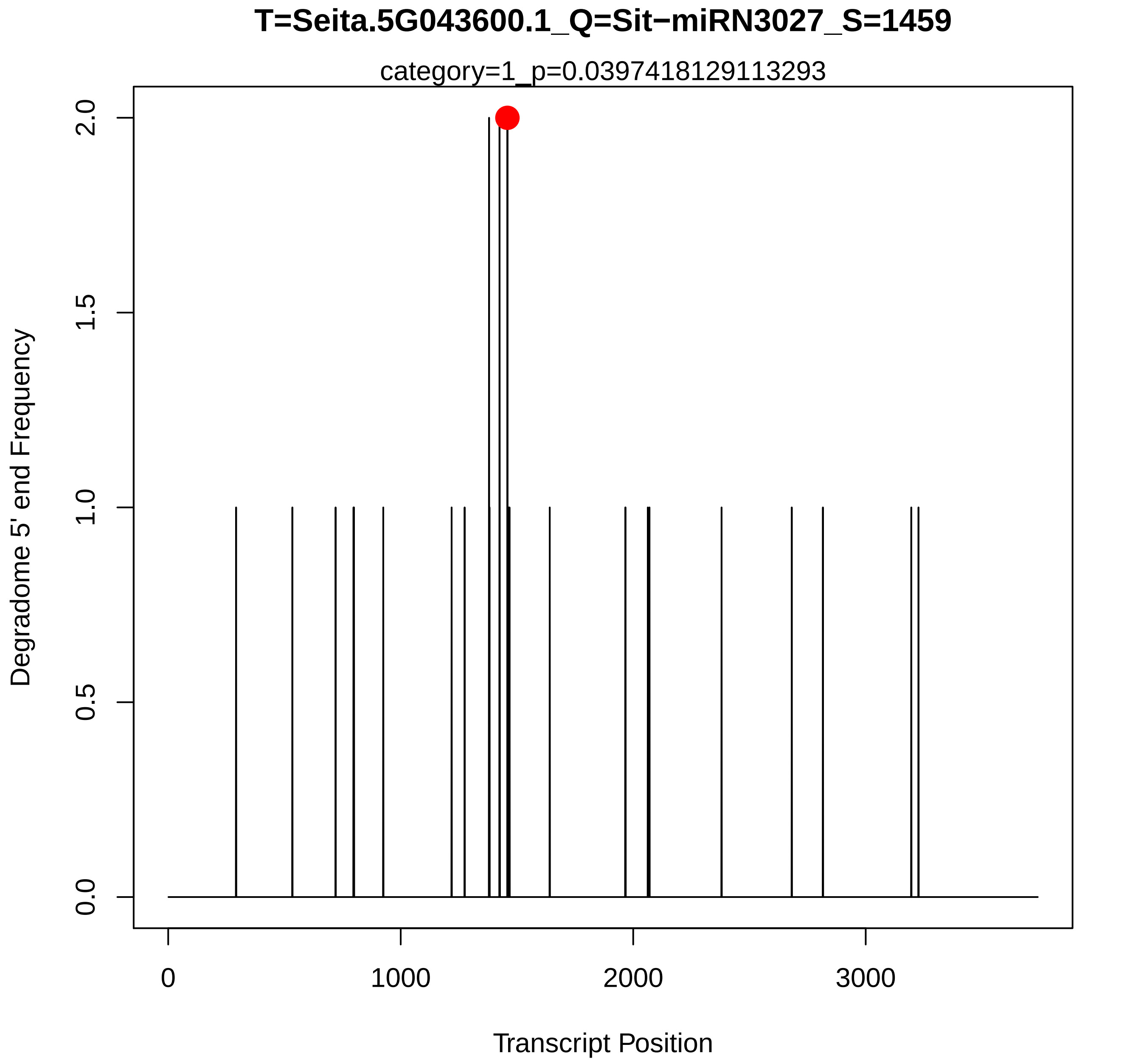
T=Seita.5G043600.1_Q=Sit−miRN3027_S=1459
category=1_p=0.0397418129113293
Degradome 5' end Frequency
Transcript Position
0.0
0.5
1.0
1.5
2.0
0
1000
2000
3000
